# Therapeutic Potential of Zeolites/Vitamin B12 Nanocomposite on Complete Freund’s Adjuvant-Induced Arthritis as a Bone Disorder: In Vivo Study and Bio-Molecular Investigations

**DOI:** 10.3390/ph16020285

**Published:** 2023-02-13

**Authors:** Amany Belal, Rehab Mahmoud, Mohamed Taha, Fatma Mohamed Halfaya, Ahmed Hassaballa, Esraa Salah Elbanna, Esraa Khaled, Ahmed Farghali, Fatma I. Abo El-Ela, Samar M. Mahgoub, Mohammed M. Ghoneim, Mohamed Y. Zaky

**Affiliations:** 1 Department of Pharmaceutical Chemistry, College of Pharmacy, Taif University, Taif 21944, Saudi Arabia; 2 Department of Chemistry, Faculty of Science, Beni-Suef University, Beni-Suef 62511, Egypt; 3 Materials Science and Nanotechnology Department, Faculty of Postgraduate Studies for Advanced Sciences, Beni-Suef University, Beni-Suef 62511, Egypt; 4 Department of Surgery, Anesthesiology and Radiology, Faculty of Veterinary Medicine, Beni-Suef University, Beni-Suef 62511, Egypt; 5 Nutrition and Food Science, College of Liberal Arts and Sciences, Wayne State University, Detroit, MI 48202, USA; 6 ZeroHarm L.C., Farmington Hills, Farmington, MI 48333, USA; 7 Biotechnology Department, Faculty of Postgraduate Studies for Advanced Sciences, Beni-Suef University, Beni-Suef 62511, Egypt; 8 Department of Pharmacology, Faculty of Veterinary Medicine, Beni-Suef University, Beni-Suef 62511, Egypt; 9 Department of Pharmacy Practice, College of Pharmacy, AlMaarefa University, Ad Diriyah 13713, Saudi Arabia; 10 Pharmacognosy and Medicinal Plants Department, Faculty of Pharmacy, Al-Azhar University, Cairo 11884, Egypt; 11 Molecular Physiology Division, Zoology Department, Faculty of Science, Beni-Suef University, Beni-Suef 62521, Egypt; 12 Department of Oncology and Department of Biomedical and Clinical Sciences, Faculty of Medicine, Linköping University, 581 83 Linköping, Sweden

**Keywords:** bone disability research, rheumatoid arthritis, bone disorders, Nano ZT/Vit B12, nanocomposites, oxidative stress, inflammation

## Abstract

Rheumatoid arthritis (RA) is a long-term autoimmune disease. As nanotechnology has advanced, a growing number of nanodrugs have been used in the treatment of RA due to their unique physical and chemical properties. The purpose of this study was to assess the therapeutic potential of a novel zeolite/vitamin B12 nanocomposite (Nano ZT/Vit B12) formulation in complete Freund’s adjuvant (CFA)-induced arthritis. The newly synthesized Nano ZT/Vit B12 was fully characterized using various techniques such as XRD, FT-IR, BET analysis, HERTEM, SEM, practical size, zeta potential, XRF, and EDX. The anti-arthritic, anti-inflammatory, and antioxidant activities as well as the immunomodulation effect of Nano ZT/Vit B12 on the CFA rat model of arthritis were examined. Histopathologic ankle joint injuries caused by CFA intrapedal injection included synovium hyperplasia, inflammatory cell infiltration, and extensive cartilage deterioration. The arthritic rats’ Nano ZT/Vit B12 supplementation significantly improved these effects. Furthermore, in arthritic rats, Nano ZT/Vit B12 significantly reduced serum levels of RF and CRP, as well as the levels of IL-1β, TNF-α, IL-17, and ADAMTS-5, while increasing IL-4 and TIMP-3 levels. Nano-ZT/Vit B12 significantly declined the LPO level and increased antioxidant activities, such as GSH content and GST activity, in the arthritic rats. In arthritic rats, Nano ZT/Vit B12 also reduced TGF-β mRNA gene expression and MMP-13 protein levels. Collectively, Nano ZT/Vit B12 seems to have anti-arthritic, anti-inflammatory, and antioxidant properties, making it a promising option for RA in the future.

## 1. Introduction

Rheumatoid arthritis (RA) is a chronic inflammatory autoimmune disease that causes synovial proliferation, progressive bony erosion, and articular cartilage degeneration [1]. Joint stiffness and tenderness after rest, as well as pain, are symptoms of RA; additional symptoms may include weakness, fatigue, and fever [2]. RA affects about 0.5–1% of the global population, with women being three times more affected than men [3]. RA usually manifests itself between the ages of 30 and 60 [4]. Although the underlying cause of RA is unknown, a number of genetic and environmental factors have been identified; nevertheless, these alone do not explain the pathogenesis [5].

Because of its similarity to human RA, CFA-induced arthritis in rats is the best model for arthritis development [6]. Chemokines and cytokines, as well as a variety of growth factors in the serum, are obviously related to the onset of RA. Cytokines are crucial in the pathogenesis of RA because they promote a number of biological processes, including inflammation. Thus, an imbalance in pro- and anti-inflammatory cytokine levels in rheumatoid joints promotes autoimmunity, resulting in joint destruction [7]. Reactive oxygen species (ROS), which are secreted into the synovial fluid by inflammatory cells as a result of the increased cytokine levels, have been identified as a significant driver of joint damage in RA patients [8].

Systemic immune suppressants are currently the only effective therapy for RA [9]. Nonsteroidal anti-inflammatory drugs (NSAIDS) are beneficial in alleviating symptoms as well as biological therapeutic approaches. Nevertheless, long-term use of these drugs has been associated with serious adverse reactions. Furthermore, these medications are costly, and not all patients respond positively to them [10]. Thus, innovative therapy with a therapeutic agent that is as efficient as traditional pharmaceuticals but without the undesirable side effects is required. Nanotherapy has now been regarded as one of the most promising technologies for the coming years. Its therapeutic approach has the potential to speed up or reverse the therapies of certain pathological conditions, and it is anticipated to have applications in alternative and integrative medicine [11]. Despite the fact that numerous drug delivery systems have been developed, strategies that have successfully made it to the clinic are uncommon. Given their diverse structures, zeolites (ZT) have attracted significant attention for controlled and targeted drug delivery purposes [12]. ZT can have microporous, mesoporous or macroporous structures, which can be used to control the delivery of diverse therapeutic agents to the target [13]. Several preclinical and clinical studies have supported the use of ZT as a novel therapeutic option for the treatment of bone abnormalities including RA [14,15]. ZT has been widely accepted as a medical device in diverse clinical attributes under European Directive 93/42/EEC [16]. Prior studies have proven that ZT can help osteoporosis patients. There have been previous reports of ZT’s beneficial effects on bone, as well as anecdotal clinician reports demonstrating improved bone density in ZT-treated individuals [17]. Several findings on bone health indicate that vitamin (Vit) B12 performs a beneficial role in the quality of human bone formation [18]. As a result, combining ZT and Vit B12 could result in a useful nanocomposite for treating RA. Accordingly, the current study aims to assess the anti-arthritic efficacy of the Nano ZT/Vit B12 in CFA-induced arthritis in rats, with a focus on anti-inflammatory and antioxidant activities, as well as on the roles of ADAMTS5, TIMP3, TGF-β, and MMPs.

## 2. Materials and Methods

### 2.1. Materials

#### Chemicals

CFA was provided by Sigma Chemical Co., St Louis, MO, USA. Vit B12 was provided by Pharma Swede Pharmaceutical Company, Cairo, Egypt. The mailing process for commercial ZT used commercial (Zeolite) as Clinoptilolite ore, Nano ZT with particle sizes ranging from 1 to 10 µm.

### 2.2. Methods

#### 2.2.1. Preparation of Nano ZT and ZT/VB12 Nanocomposite

**Figure 1:**
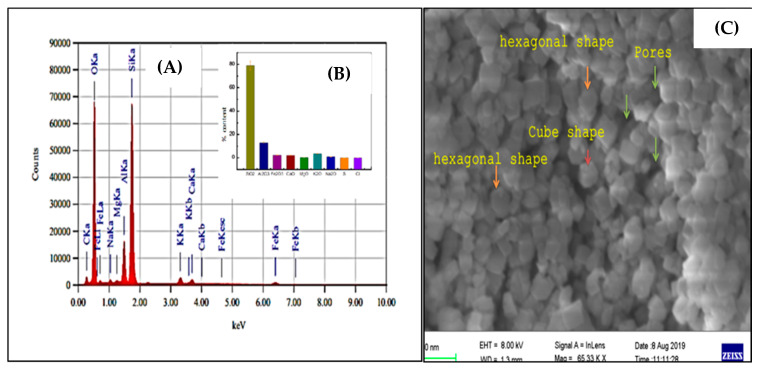
EDX analysis (**A**,**B**); the inset figure represents the XRF and the SEM (**C**) for the prepared Nano ZT.

The Nano-ZT was created by passing commercial ZT through processed natural clay ZT firstly activated through dryer at 100 °C to 48 h in a photon ball milling vessel for 12 h at a continuous mechanical speed of 300 rpm under the conditions listed in Table S1. EDX and XRF techniques were used to identify the content element (Figure 1A–C).

For the synthesis of Nano ZT/Vit B12, Vit B12 solution (1000 ppm) was dissolved in DDW water/dimethylsulfoxide (DMSO) (1:1 volume %). Then, Nano ZT (300 mg) was added into the Vit B12 solution (50 mL), and it was stored in the dark for 24 h at RT. The obtained suspended solution was centrifuged at 130,000 rpm for 30 min. The Vit B12-loaded Nano ZT particles were washed with DDW water and dried under vacuum for 12 h at 60 °C. The Vit B12 solution and its supernatant were collected. The concentration of Vit B12 in the solutions was measured by UV–viz spectrophotometrically at 360 nm to determine the Vit B12 loading efficiency in Nano ZT [19]. The solution was filtered after centrifugation by using 0.45 mm nylon filters before spectrophotometric analysis, as shown in Figure S2. The loading efficiency percent (LE %) was calculated using Equation (1):

(1) $$\mathrm{LE}\%=\;100*\left(\mathrm{amount}\;\mathrm{of}\;\mathrm{VB}12\;\mathrm{incorporated}\;\mathrm{in}\;\mathrm{Nano}\;\mathrm{ZT}\right)⁄\left(\mathrm{Intial}\;\mathrm{concentration}\;\mathrm{of}\;\mathrm{VB}12\right)$$

The Vit B12 loading capacities of NZ in mg/g were calculated from Equation (2)

Loaded drug (mg/g) = (initial concentration − residual concentration) × solvent volume(2)

carrier weight amount of Vit B12 incorporated in Nano ZT is the initial concentration of Vit B12, the concentration after equilibrium time, solvent volume is the solvent volume in ml, and carrier weight is the NZ weight in g. The experiment results were considered for the tests in triplicate form with a standard deviation of less than 3.7%.

#### 2.2.2. Nano ZT/Vit B 12 Characterization

The formed Nano ZT and Nano ZT/Vit B 12 were characterized by XRD (PANalytical Empyean, Sweden). An accelerating voltage of 40 KV was applied, using scan angle ranging from 5 to 80°, a scan step of 0.05°, and a 30 mA current. A Bruker devise (vertex 70 FTIR-FT Raman) was used to investigate the vibration of the chemical bonds. A Germany spectrophotometer (serial number 1341) screening the frequency range of 400–4000 cm^−1^ was applied using a potassium bromide disc. The surface morphology of the prepared materials was investigated using a scanning electron microscope (SEM), Germany. EDX (Quanta FEG250, Germany) was performed to determine the elemental composition in the synthesized materials. The BET specific surface area, pore volume and pore size distribution of the Nano ZT were estimated by N_2_ adsorption–desorption method by an automatic surface analyzer (TriStar II 3020, Micrometrics, USA). High-resolution transmission electron microscopy (HRTEM) (JEOL-JEM 2100) was applied to determine the microstructures of the produced materials. XRF analysis was performed on the natural Nano ZT to confirm the structure using XRF-ARL-9900. The partial size and zeta potential were investigated [20].

### 2.3. Molecular Simulation Calculations

Monte Carlo (MC) and quench dynamics simulations were performed, respectively, using the Adsorption Locator and Forcite modules implemented in the BIOVIA Materials Studio 2020 package [https://www.3ds.com/products-services/biovia/products/molecular-modeling-simulation/biovia-materials-studio/ accessed on 7 February 2023]. The structure of zeolite (HUE) was taken from the material studio database. The Adsorption Locator was used to find low-energy adsorption sites of Vit B12 on the HUE (0 0 1) surface using the Metropolis Monte Carlo searches of the system, as the temperature decreased according to a simulated annealing method, where the number of cycles was 5 and steps per cycle was 50,000. The maximum and final temperatures were 1.0 × 10^5^ and 100.0 K, respectively. The Bravais lattice of the HUE structure was centered monoclinically. The Vit B12 and HUE were optimized using the Forcite module. The optimized angles α, β, and γ were 108.297°, 108.297°, and 89.5576°, respectively, while the optimized lattice lengths of A, B, and C are 12.5560, 12.5560, and 7.47176 Ǻ, respectively. A HUE (0 0 1) surface was cleaved from the optimized HUE, with 3 U × 3V, and 40 Ǻ vacuum above the surface. In both simulations (MC and quench dynamics) and the optimization process, the COMPASSIII force field and its charges were used [21]. The electrostatic interaction term was calculated using the Ewald method, with 1.0 × 10^−4^ kcal/mol Ewald accuracy and 15.5 Ǻ cutoff distances. An atom-based method was used to calculate the van der Waals, and the truncation method was cubic spline with 15.5 Ǻ cutoff distance and 1 Ǻ spline width. The lowest-energy structure obtained from the MC simulation was used as the input structure of the quench dynamics simulation. The quench dynamics simulation combines minimization and dynamics to find the lowest-energy structures. The dynamic setup was as follows: NVT ensemble, 298.0 K (controlled by Nose thermostat), 1.0 *fs* time step, 1000 ps simulation times = 1000 ps (1.0 × 10^6^ steps), and quench every 5000 steps. In the optimization step, the smart algorithm was used, and the convergence tolerance of the energy, force, stress, and displacement was 1.0×10^−4^ kcal/mol, 0.005 kcal/mol/Ǻ, 0.005 GPa, and 5.0 × 10^−5^ Ǻ. The interaction was calculated for the obtained lowest energy structure of the system by using the following equation:

$$E_{\mathrm{interaction}}={\;E}_{\mathrm{total}}-\left(E_{\mathrm{surface}}+E_{\mathrm{Vit}\;B12}\right)\;$$

$E_{\mathrm{total}}$ is the energy of the zeolite surface and Vit B12, $E_{\mathrm{surface}}$ is the energy of the zeolite surface without Vit B12, and $E_{\mathrm{Vit}\;B12}$ is the energy of Vit B12 without the zeolite surface.

### 2.4. Experimental Animals

The Egyptian Organization for Biological Products and Vaccines (VACSERA) Helwan, Cairo, Egypt, provided 30 male Wistar rats weighing 120–150 g. Before beginning the experiment, the animals were closely monitored for two weeks to rule out any concurrent infections. Rats were housed in precisely calibrated polypropylene cages and kept in climate-controlled environments with humidity levels around 55.5%, RT of 22 °C, and a 12 h light–dark cycle. The animals were given an unlimited supply of rat chow and water to drink. The experiment was carried out at Beni-Suef University’s Faculty of Veterinary Medicine in accordance with the principles and guidelines of the Canadian Council on Animal Welfare and Ethics for Regional Experimental Animals at Cairo University, Egypt.

### 2.5. Induction of Arthritis

To induce arthritis, 0.1 mL of CFA solution was intrapedally injected into the right hind paw footpad once per day for two days [22].

### 2.6. Animal Grouping

**Figure 2:**
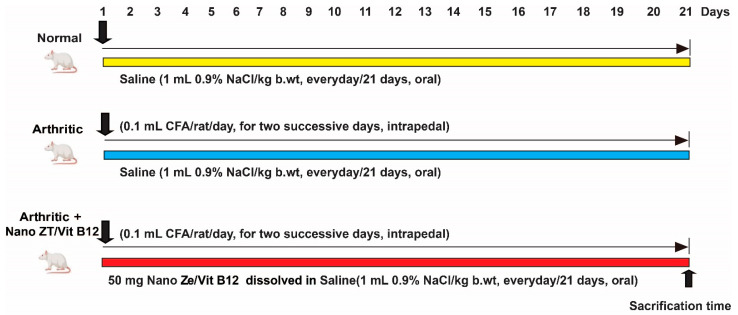
Experimental design and animal grouping.

Following the induction of RA with CFA, 30 Wistar rats were split into three different groups of ten rats each, as shown in Figure 2.

Group 1: (Normal control rats): this group was given the equivalent amount of vehicle (saline) orally every day for 21 days.

Group 2: (Arthritic control group): CFA-induced arthritis was treated for 21 days with equal amounts of saline taken orally every day, just as in the control group.

Group 3: (Arthritic + Nano ZT/Vit B12): CFA-induced arthritis was given a safe oral dose of Nano ZT/Vit B12 at a daily dose of 50 mg/kg b.wt [23] for 21 days.

### 2.7. Assessment of Paw Edema

Paw volume is measured in all groups to track the progression of arthritis by assessing paw edema and swelling rate. Before being measured, rats were put to sleep by inhaling a 1:2:3 ACE mixture of alcohol, chloroform, and ether. Day 0 was the first day of CFA injection, and measurements were taken on days 3, 7, 14, and 21 after the arthritis induction. The volume of the hind paw was calculated using an electronic caliper.

### 2.8. Histopathological Examination

For the study, rats were slaughtered on the 21st day after arthritis induction, and the posterior ankle joints of the right leg of four rats from each group were removed and placed in 10% buffered formalin for 48 h. Then, 10% formic acid was used to decalcify the bones. For two weeks, the solution was changed twice per week, and a surgical blade was used to determine when the decalcification process was complete. Following the decalcification process, samples were washed with phosphate buffer saline (PBS), dried with a graded series of ethanol, and embedded in paraffin wax cubes. After that, 5 mm thick sagittal slices were created and stained with hematoxylin and eosin (H&E). A blind histological examination was performed by a center for pathology including synovitis, cartilage and bone damage. Sections were classified for cartilage degeneration, bone erosion, synovial hyperplasia (pannus development), and inflammation (infiltration of mononuclear cells) using the system described by Sancho et al. [24]. Each characteristic was rated from 0 to 2, with 0 representing normal, 1 (+) representing mild inflammation, 2 (++) representing moderate inflammation, and 3 (+++) representing severe inflammation. Blood vessels, inflammatory infiltrates, articular cartilage, pannus, and menisci were found in the joint cavity space.

### 2.9. Biochemical Investigations

The levels of LPO and GSH, as well as GST activity, were measured in the liver homogenate using the following methods of Preuss et al. [25], Beutler et al. [26], Matkovics et al. [27], and Manervik and Gutenberg [28], respectively. RF, CRP, 1L-1β, 1L-17, 1L-4, TNF-α, ADAMTS-5, and TIMP-3 levels of serum were determined utilizing particular ELISA kits, according to the manufacturer’s instructions.

### 2.10. Western Blot Analysis

MPP13 expression was evaluated in rat ankles using Western blot, as previously described [29]. In RIPA buffer, the frozen ankle joint samples were homogenized with a phosphatase/proteinase inhibitor cocktail. Homogenate was then centrifuged, and the protein concentration was determined using the Bradford reagent. SDS-PAGE 10% gels were used to separate equal amounts of proteins, which were then transferred to PVDF membranes. After blocking with 5% BSA, the membranes were incubated overnight at 4 °C with primary antibodies against MPP13 and β-actin (Santa Cruz Biotechnology, Inc., Dallas, TX, USA). The membranes were washed and then incubated for 1 h at RT with the appropriate secondary antibody. A CCD camera-based imaging device was used to capture chemiluminescent signals. On a ChemiDoc MP imager, image analysis software was used to read the band intensities of the target proteins against the β-actin by protein normalization.

### 2.11. q-RT-PCR Analysis

After extracting total RNA from the tissue with Trizol, the quantity and quality of RNA were determined using a Beckmann dual spectrophotometer. Reverse transcription to cDNA was carried out using a reverse transcription kit (Applied Biosystems, Foster City, CA, USA). SYBR Green Master Mix was used to amplify the cDNA (ThermoFisher, USA). Using the 2−ΔΔCT method, the expression of the TGF-β was normalized to the expression of rat GAPDH. Table 1 lists the primers used in the experiments.

### 2.12. Statistical Analysis

Statistics for the Social Sciences, version 22, was used for the statistical analysis (SPSS, Chicago, IL, USA). To compare the means of several groups, a one-way analysis of variance (ANOVA) was used, followed by Tukey’s post multiple comparison test. The values are denoted by the mean ± SEM. At *p* < 0.05, the result was statistically significant.

## 3. Results

### 3.1. Nano Synthesis and Characterization

**Figure 3:**
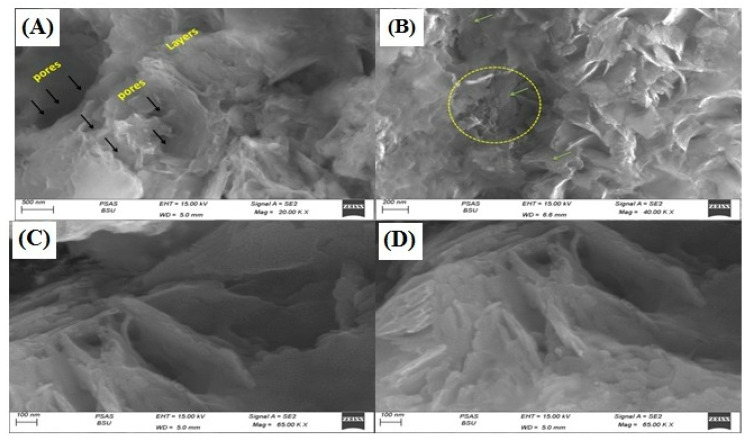
SEM images of prepared Nano ZT/Vit B12 (**A**–**D**).

In this current study, we chose Nano ZT as our Vit B12 carrier owing to its facile preparation, ordered structure, and uniform particle size and shape besides its efficient role in biomedical application. The mailing process was also expected to improve the loading efficiency of Vit B12. The particle morphology was determined by SEM. Figure 3A–D show the SEM images, which give information about the morphological structure of the Nano ZT/Vit B12. The natural Nano ZT/Vit B12 was exhibiting rough layers of morphology with a number of pores and cavities suggesting the higher porosity. The Vit B12 was loaded onto the ZT surface, which clearly appear in (Figure 3C). The hollow cores and mesoporous shells that present within the prepared materials ZT (Figure 3D) provide great space for Vit B12 storage. The layer structure with high porosity and homogeneity, giving the chance for enhancing the biological activity. The zeta potential was −13.5 for Nano ZT and −30 for Nano ZT/Vit B_12_, from the data presented in Figure S1, we can conclude that increases to a more negative in the value of zeta potential conform the loading of Vit B_12_ on the surface of the Nano ZT.

**Figure 4:**
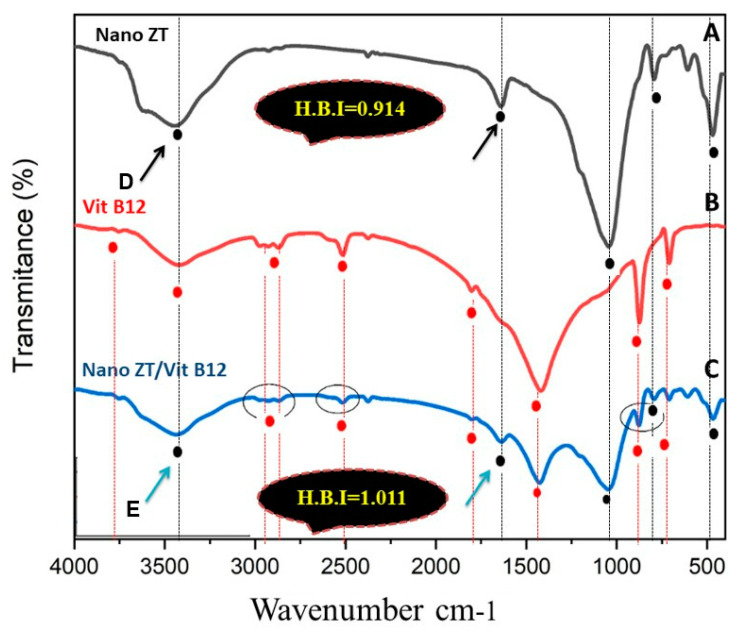
FTIR of the Nano ZT, VB12 and Nano ZT/Vit B12 (**A**–**C**); XRD pattern of Nano ZT (**D**) and Nano ZT/Vit B12 (**E**).

Figure 4A–C shows the FTIR spectra of Nano ZT, Vit B12, and Nano ZT/Vit B12 where at 3700–1600 cm^−1^, it was related to hydrated Nano ZT (A). A broad band was at about 3440 cm^−1^, which can assign hydrogen-bonded OH attached with oxygen ions and Al ions; a broad band at about 1640 cm^−1^ is the usual bending vibration of (O-H) in water molecules. Bands in the region 1100–800 cm^−1^ were attributed to the internal tetrahedron vibrations of Si-O-Si and Si-O-Al. The FTIR spectrum of Nano ZT/ Vit B12 is presented in Figure 4C. Signed Spectra of Vit B12 (Figure 4B) were at 3753.79, 3421.63, 2973–2870, 2516.20, 2374.49, 1801.84, 1419.14, 873.93 and 709.37 cm^−1^. The band assigned at 2973–2870 cm^−1^ is characteristic of stretching of C-H, and the band assigned at 1801.84 cm^−1^ is characteristic of stretching of C=C, 1419.14 cm^−1^ for C=O and 873.93 and 709.37 cm^−1^ for C-O. Decreasing the intensity of some peaks related to Vit B12 in the Nano ZT/Vit B12 could conform the loading of Vit B12. In addition, the peak’s broadening at 3421.63 cm^−1^ confirms an increase in H-Bond. Moreover, intramolecular H-bonding over the prepared nanocomposites was performed through calculating the intensity of the hydrogen bonding from the ratio of the absorbance bands at 3447 and 3436.59 cm^−1^ (for the –OH peak) and 1639.90 and 1636.95 cm^−1^ (for the O-H peak) in Nano ZT and Nano ZT/Vit B12, respectively, showing a significant increase in the case of the Nano ZT/Vit B12 nanocomposite (1.011) compared to that of Nano ZT (0.914).

**Figure 5:**
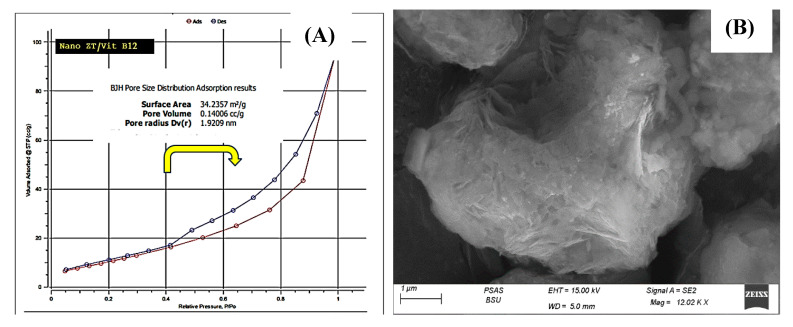
(**A**,**B**) N_2_ adsorption–desorption isotherms and SEM of Nano ZT/Vit B12.

The powder XRD patterns of Nano ZT and Nano ZT/VB12 are shown in Figure 4D–E. It is clear that all prepared samples keep the typical diffraction peaks of the Nano ZT structure; notably, the intensity of the diffraction peaks of the samples gradually decreases and the other peaks increase, indicating that VB12 could enter into the ZT framework of the Nano ZTs. Surface area analysis of the prepared materials was performed to understand the specific surface area and their porosity. N_2_ sorption was measured, as shown in Figure 5A, which displays the N_2_ adsorption−desorption isotherm. The curves show that the Nano ZT/Vit B12 follows type IV, according to the International Union of Pure and Applied Chemistry (IUPAC) shape. In addition, there is a closed adsorption−desorption hysteresis loop with a value of pressure from 0.40 to 1.00 that is related to capillary condensation and mesopores was observed. This result is consistent with the SEM observations (Figure 5B). Moreover, the results are supported by the wide distribution of pore size in Table S2, resulting from the hierarchical structure of ZT. The BET specific surface area of the nanoparticles is 34.2357 m^2^/g, and the maximum pore volume is about 1.4006 cc/g nm. This specific surface area results from the porous structure of the prepared nanoparticles (Figure 5A). Thus, we proposed that such a structure may be favorable for medical applications, as there are several preclinical and clinical investigations that have supported the use of ZT as a unique therapeutic choice for the treatment of bone abnormalities, including RA [14,15,16,17].

### 3.2. Molecular Simulations

**Figure 6:**
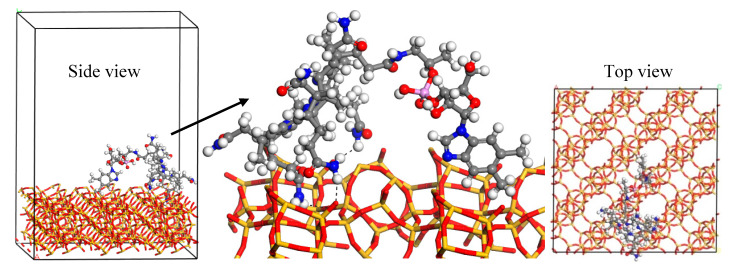
The lowest-energy structure of the adsorbed Vit B12 on the (001) Nano ZT surface, as obtained from the quench dynamics simulation. The atom colors: silicon (yellow), oxygen (red), nitrogen (blue), carbon (grey), phosphorus (pink), and hydrogen (white). The dashed black lines are the hydrogen bonds.

Monte Carlo and quench dynamics simulations were used to know how Vit B12 was loaded onto the Nano ZT nanoparticles. MC simulation confirmed that Vit B12 was unable to be located in the Nano ZT pores due to its large size. Therefore, the Vit B12 adsorption on the (001) Nano ZT surface was searched for. The obtained lowest-energy structure was further simulated by quench molecular dynamics to sample many different low-energy configurations. The interaction energy was −97.8 kcal/mol, indicating the preferential adsorption of Vit B12. The lowest-energy structure of the adsorbed Vit B12 on the Nano ZT surface is shown in Figure 6. The main interactions were hydrogen bond formation between the amine group and the oxygen atoms of the Nano ZT structure.

### 3.3. Effect of Nano ZT/Vit B12 on Gross Lesions of the Paw and Ankle Joint

**Figure 7:**
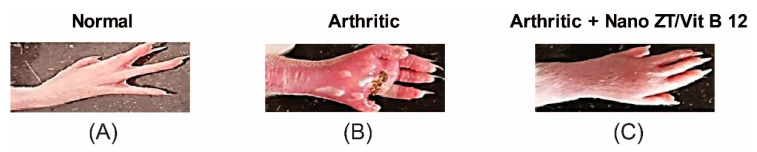
The gross morphology of rats’ right hind paws and ankles reveals swelling and inflammation in rats of different groups. (**A**) Normal rats, (**B**) arthritic rats, and (**C**) arthritic rats received Nano T/Vit B 12.

The right hind paws and ankles of CFA-induced arthritic rats showed swelling, edema, and redness, which appear to be outer markers of arthritis symptoms. These visible lesions were significantly reduced in the arthritic groups that received Nano ZT/Vit B 12 (Figure 7).

### 3.4. Effect of Nano ZT/Vit B12 on Right Hind Paws Volume

**Figure 8:**
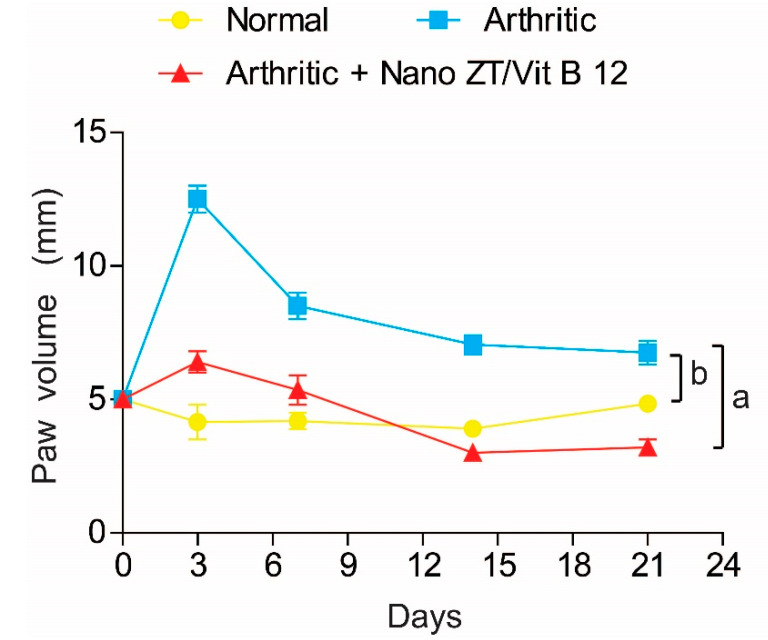
Effect of Nano ZT/Vit B 12 on right hind paw size in rats with CFA-induced arthritis. Means, which have different symbols, differ significantly at *p* < 0.05.

Rats with CFA-induced arthritis had a significant (*p* ˂ 0.05) increase in paw size (edema) when compared to the control group. In contrast, the Nano ZT/Vit B12-treated rats had smaller paws when compared to the arthritis group (Figure 8). Paw edema in different groups was measured using digital calipers, as was the circumference of the right hind leg in the paw region as a measure of swelling velocity.

### 3.5. Effect of Nano ZT/Vit B12 on Serum RF, CRP, TNF-α, IL-1β, IL-17, ADAMTS-5, IL-4, and TIMP3 Levels

**Figure 9:**
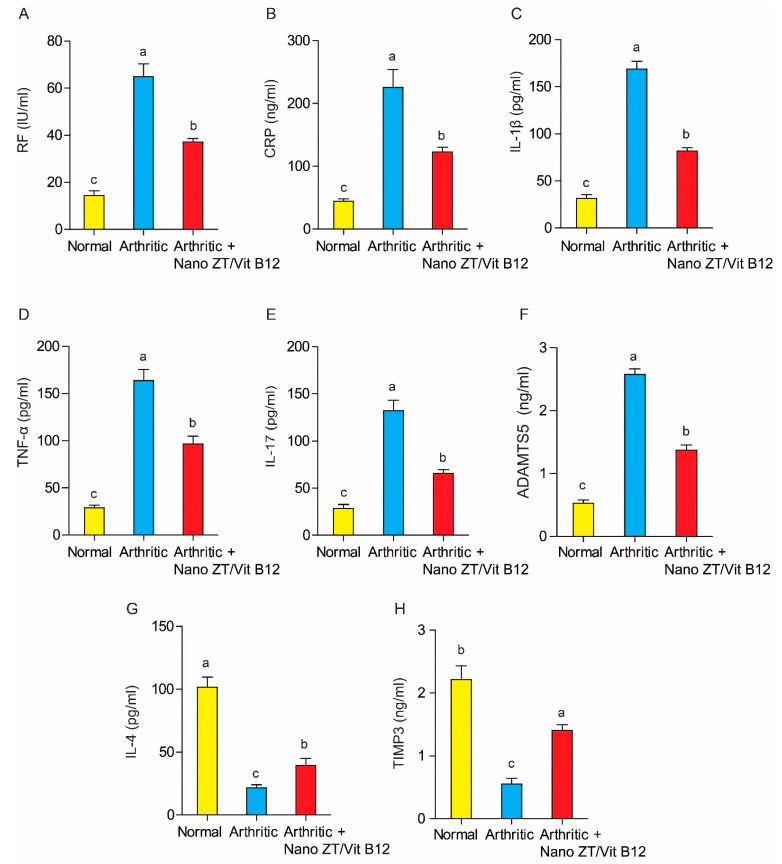
Effect of Nano ZT/Vit B12 on serum RF (**A**), CRP (**B**), IL-1β (**C**), TNF-α, (**D**) IL-17 (**E**), ADAMTS5 (**F**), IL-4 (**G**), and TIMP3 (**H**) levels. Data are presented as mean ± SEM; the values for each parameter indicated with a different superscript alphabet (s) (a, b, and c) are significantly different (*p* < 0.05).

The effect of Nano ZT/Vit B12 on RF, CRP, TNF-α, IL-1β, IL-17, ADAMTS-5, IL-4, and TIMP3 levels is depicted in Figure 9. CFA administration to rats resulted in a significant increase (*p* ˂ 0.05) in serum levels of RF, CRP, TNF-α, IL-1β, IL-17, and ADAMTS-5, as well as a significant decrease (*p* ˂ 0.05) in serum levels of IL-4 and TIMP3. When arthritic rats were given Nano ZT/Vit B12, their serum levels of RF, CRP, TNF- α, IL-1β, and IL-17 decreased significantly (*p* ˂ 0.05) when compared to the arthritis group. The Nano ZT/Vit B12 treatment, on the other hand, significantly increased serum levels of IL-4 and TIMP3.

### 3.6. Effect of Nano ZT/Vit B12 on LPO, GSH Content, and GST Activity

**Figure 10:**
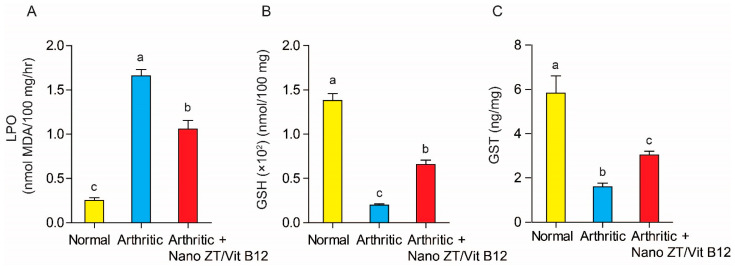
Effect of Nano ZT/Vit B12 on hepatic LPO (**A**) and GSH content (**B**) as well as GST activity (**C**). Data are presented as mean ± SEM; the values for each parameter indicated with a different superscript alphabet (s) (a, b, and c) are significantly different (*p* < 0.05).

As illustrated in Figure 10. When arthritic rats were compared to normal control rats, hepatic LPO was significantly (*p* ˂ 0.05) increased. In contrast, treatment with Nano ZT/Vit B12 resulted in a significant (*p* ˂ 0.05) reduction in elevated hepatic LPO. As a result, Nano ZT/Vit B12 appears to be more effective in improving LPO in osteoporotic rats. Furthermore, when compared to normal control rats, CFA administration resulted in a significant (*p* ˂ 0.05) decrease in hepatic GSH content as well as GST activity. However, the hepatic GSH content and GST activity were significantly improved (*p* ˂ 0.05) after treatment with Nano ZT/Vit B12.

### 3.7. Effects of Nano ZT/Vit B12 on TGF-β mRNA Expression and MMP13 Protein Levels

**Figure 11:**
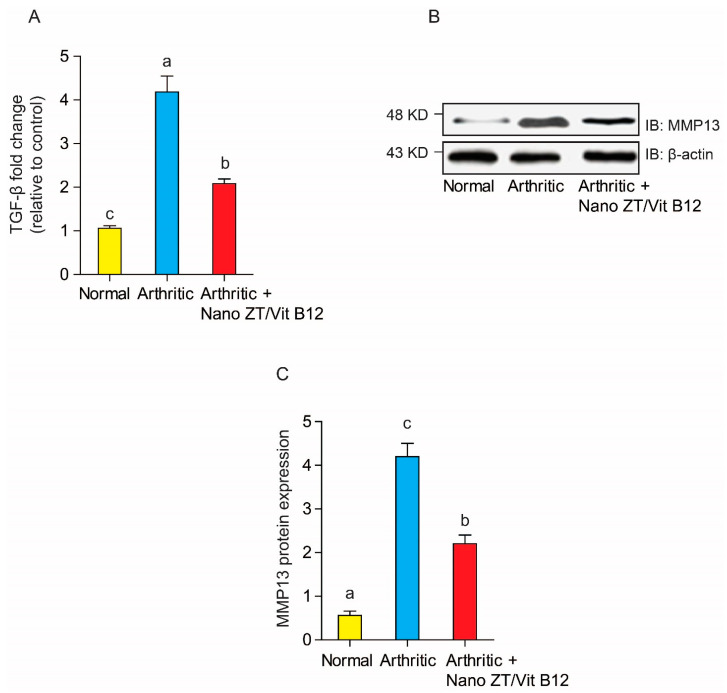
Effects of Nano ZT/Vit B12 (**A**–**C**) on TGF-β mRNA expression (**A**) and MMP13 protein levels (**C**). Data are presented as mean ± SEM; the values for each parameter indicated with a different superscript alphabet (s) (a, b, and c) are significantly different (*p* < 0.05).

TGF-β mRNA expression was significantly (*p* < 0.05) higher in arthritic rats than in the control group, as shown in Figure 11. TGF expression, on the other hand, was significantly (*p* < 0.05) reduced in the Nano ZT/Vit B12-treated group. Similarly, when rats were given CFA, the protein level of MMP-13 increased significantly (*p* < 0.05) when compared to normal control rats. Nonetheless, MMP-13 protein levels in arthritic rats were significantly reduced (*p* < 0.05) after treatment with Nano ZT/Vit B12.

### 3.8. Histopathological Changes and Arthritic Score

**Figure 12:**
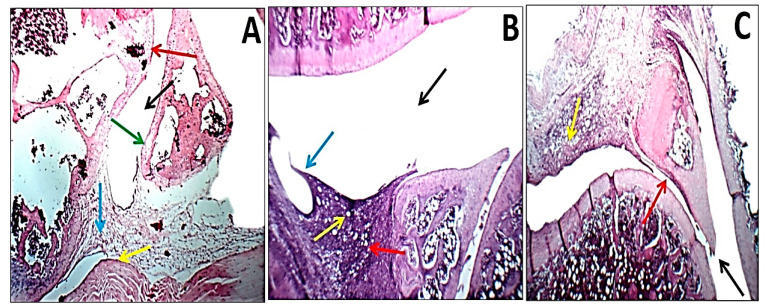
Photomicrographs of H&E-stained sections illustrating the impact of Nano ZT/Vit B12 on histopathologic changes in the ankle joints of arthritic rats. In normal control rats (**A**), the joint shows the average joint cavity (black arrow), average synovial lining (blue arrow), average sub-synovial tissue (yellow arrow), average articular cartilage with intact superficial layer (red arrow), and average menisci (green arrow). Joints of arthritic rats (**B**) show an average joint cavity (black arrow), destructed pannus on the menisci (red arrow), villous formation of synovial lining (blue arrow), and a significant sub-synovial inflammatory infiltrate (yellow arrow). In arthritic rats given Nano ZT/Vit B12 (**C**), the joints had an average joint cavity (black arrow), a small pannus on the menisci (red arrow), and a mild sub-synovial inflammatory infiltrate (yellow arrow) (H&E X 100).

When sections of the right ankle joint of the hind leg of normal control rats were stained with H&E, there was no inflammation. The synovium of rats with CFA-induced arthritis, on the other hand, showed hyperplasia as well as significant infiltration of inflammatory cells and extensive cartilage deterioration, while rats given Nano ZT/Vit B12 had nearly normal articular surfaces and synovial membranes with no signs of inflammation (Figure 12). In addition, Table 2 shows the histological lesion scores of the joint damage.

## 4. Discussion

In the current study, since the ZT materials (Figure 1C) have pores and cavities that can act as channels for regulating the delivery of Vit B12, they can act as Vit B12 transporters via hydrogen bonding interactions [30]. Furthermore, the significant increase in particle size and the change in hydrodynamic size diameters of the ZT from 420.70 to 640.60 nm for ZT/Vit B12 confirmed the loading of Vit B12 onto the surface of the Nano ZT (Figure S1). Furthermore, the H-bond intensity in Nano ZT is 0.86, while in ZT/Vit B12, it is 0.953, indicating that Vit B12 was successfully loaded onto the surface of the Nano ZT. In addition to the FTIR, surface texture analysis and the XRD powder patterns of Nano ZT and ZT/Vit B12 samples shown in Figure 4C–D, it is clear that all as-prepared samples retain the typical diffraction peaks of the Nano ZT structure [31], whereas the intensity of the diffraction peaks gradually decreases and increases due to the incorporation of Vit B12 into the Nano ZT [32].

In order to explore the therapeutic potential of ZT/Vit B12 on CFA-induced arthritis, an in vivo arthritis model was established. To evaluate the potential effect of the anti-inflammatory drug RA, the alteration in paw edema has traditionally been used [33]. Our findings revealed that when arthritic rats were given Nano ZT/Vit B12, their increased hind paw size was reduced compared to arthritic rats. This decrease in paw size reveals a reduction in swelling rate, which can be thought to be due to the suppression of inflammatory processes [34]. These insights may be believed to be due to the anti-inflammatory properties of Nano ZT/Vit B12.

Moreover, autoantibodies are produced in serum and synovial fluid (SF) samples in 50–80% of RA patients, making RA a widely known autoimmune disease [35]. The presence of RF, a circulating IgG antibody, is a significant serum marker for RA diagnosis and prognosis [36]. RF is produced by B lymphocytes, indicating the joint immune complexes formation [37]. In RA, CRP is evaluated as an indicator of systemic inflammation. It is, nevertheless, an immune regulator that is involved in the inflammatory pathways associated with RA and that promotes atherogenic effects [38]. In parallel with Shaban et al. [39], the serum levels of RF and CRP were significantly higher in the arthritic rats. However, supplementing arthritic rats with Nano ZT/Vit B12 significantly reduced both RF and CRP serum levels, indicating that Nano ZT/Vit B12 has an anti-inflammatory effect. Cytokines play an important role in the disease’s etiology. RA is distinguished by the constant infiltration of immune cells (monocytes and lymphocytes) into the joints [40]. Proinflammatory cytokines such as TNF-α, IL-17, and IL-1β have been shown to be activated, contributing to long-term inflammation and bone loss. They are also inflammatory mediators, which are used to initiate and sustain inflammation [41]. Anti-inflammatory mediators, also known as anti-inflammatory cytokines, are used to stop the process (IL-4) [42]. The imbalance between the two mediators causes cell damage, cartilage and bone destruction, and inflammation in chronic inflammatory conditions [42]. It has been reported that IL-1β is the most important cytokine in the development of pathogenic arthritis and has been linked to symptoms such as morning stiffness. This cytokine is primarily produced by macrophages and is involved in inflammatory cell invasion as well as in cartilage and bone degeneration [43]. Furthermore, it enhances the differentiation of osteoclasts through the activator of nuclear factor B ligand receptor B on macrophages, which absorbs and damages bone [43]. In addition, IL-1β stimulates the synthesis of prostaglandins (PGE-2), matrix metalloproteinases (MMP), and inducible nitric oxide (iNOS), all of which contribute to cartilage degradation [44]. In contrast, the anti-inflammatory cytokine IL-4, which is generated by Th-2 helper cells, reduces IL-1β and TNF-α production while also preventing cartilage injury. Furthermore, IL-4 has been shown to reduce IL-1β production and increase the expression of its receptor antagonist, both of which reduce inflammation in RA synovial samples [45]. Because of its protective effect in RA mouse models, IL-4 has the potential to be used as a treatment for autoimmune diseases [46]. Consistent with all of our previous findings, the arthritis control group revealed a significant increase in serum levels of IL-1β, TNF-α, and IL-17 and a significant reduction in the levels of IL-4 in arthritic rats. Our results are in harmony with Shaban et al. [39]. These results are a clear indication that CFA injections favor the inflammatory pathway over anti-inflammatory therapy. However, rats given Nano ZT/Vit B12 demonstrated a significant shift toward the anti-inflammatory pathway and Th-2 dominance over Th-1, as evidenced by lower levels of IL-1β, TNF-α and IL-17 and higher levels of IL-4, indicating that Nano ZT/Vit B12 has an anti-inflammatory effect.

MMPs are necessary for tissue remodeling in a broad range of biological processes including angiogenesis, embryogenesis, morphogenesis, and wound healing. While basic biological events such as pregnancy and wound healing occur, MMP expression and activity fluctuate [47]. MMP-3 performs a crucial role in the pathogenesis of ankylosing spondylitis and RA. Assessing the levels of active MMP-3 in clinical samples may reveal information about the progression of rheumatic diseases and their promising therapeutic responses. In order to accurately measure the active form of MMP-3 (act-MMP-3) in both ex vivo and human serum models, researchers set out to develop a sensitive assay test [48]. Our findings showed that MMP-13 protein levels were upregulated in arthritic rats, which is consistent with the findings of Shaban et al. [39]. However, rats given Nano ZT or Vit B12 had lower levels of MMP-13 protein, indicating that Nano ZT/Vit B12 has an anti-inflammatory effect against RA. An additional role in inflammation was revealed as a result of the identification of novel metalloproteinase substrates in this environment. These proteases now act as mediators of inflammatory signals involving various chemical compounds and cytokines as well as enzymes that remodel the extracellular matrix. TIMPs have the ability to control the inflammatory response and have an effect on conditions such as RA because they are naturally occurring inhibitors of these metalloproteinase. TIMP-3 stands out as an important regulator of inflammation because of its ability to accurately block proinflammatory cytokines and joint tissue damage [49]. In our study, the Nano ZT/Vit B12 treatment significantly increased TIMP-3 levels as joint anti-inflammatory markers in RA-induced rats. Since several studies have confirmed the presence of TGF-β in the synovial tissues and synovial fluids of patients with RA, it has been suggested that TGF-β plays a role in the pathogenesis of RA [50]. TGF-β controls the activity of fibroblasts [51]. In this work, we considered the expression of TGF-β receptors in the synovial membrane of the ankle joint of RA rats and demonstrated the importance of synovial fibroblast functional responses to TGF-β in this condition, which is significantly reduced after treatment with the Nano ZT/Vit B12. In fibroblasts from RA patients, TGF-β increased connective tissue growth factor (CTGF) production more than in osteoarthritis (OA) patients. TGF-β significantly reduced the proliferation of RA synovial fibroblasts and enhanced their chemotactic migration [52]. Thus, the Nano ZT/Vit B12 played an important role in the downregulation of TGF-β. Our results are in parallel with previous research on the use of zeolite in the treatment of orthopedic diseases [12]. Normal adult human osteoblast-like cells produced more transformed growth factor (TGF) and proliferated and differentiated in vitro. In addition, it causes normal human osteoblast-like cells to synthesize more DNA in a dose-dependent manner [53]. Alkaline phosphatase activity and steocalcin release are also elevated by zeolite [12]. TGF is a powerful osteoblast mitogen. The latent form of TGF protein is released into the conditioned medium 6 h after receiving zeolite A treatment, which also raises steady-state mRNA levels of TGFh1. Therefore, zeolite was used in a previous work to stimulate the proliferation and differentiation of osteoblast lineage cells [54]. Its therapeutic benefit in individuals with osteoporosis is due to its ability to stimulate bone formation because of its safety, which was assessed in previous toxicological studies [23].

As markers of the inflammatory joint, disintegrin and metalloproteinase with thrombospondin motifs (ADAMTS-5) are implicated in the pathophysiology of RA [55]. ADAMTS-5, the primary aggrecanase-degrading articular cartilage matrix, has been proposed as a therapeutic target for RA [55]. An updated understanding of ADAMTS-5 control such as the recently found treatment approaches for RA was provided by previous investigations [56]. In the present study, Nano ZT/Vit B12 exhibited a significant reduction in ADAMTS-5 in arthritic rats. Additionally, in this study, Vit B12 and ZT were mixed because Vit B12 is essential for bone health. The role of Vit B12 in the quality of human bone formation has been demonstrated in some research on promoting bone health [57]. Serum Vit B12 levels in adults have been linked to biochemical markers and pro-inflammatory cytokines [58].

Maintaining adequate Vit B12 levels may reduce inflammation through antioxidant activity, which cells use to defend themselves against the destructive effects of free radicals [59]. Anti-inflammatory cytokines and other inflammatory byproducts are increased as a result of antioxidant depletion. In addition, the majority of RA patients were shown to have multiple preexisting anemic factors at the same time as moderate Vit B12 deficiency [59].

Oxidative stress is an important component of RA disease physiology that should not be overlooked. Previous research by Holly and Cheeseman [60] and Ali et al. [61] demonstrated how the accumulation of granulocytes and macrophages in an inflammatory area promotes the production of free radicals and ROS, such as superoxide (O_2_) and hydrogen peroxide radicals (H_2_O_2_). Reactive oxidants are produced in a variety of cell compartments, either naturally through endogenous metabolism or as a result of external noxious or harmful stimuli [62,63]. Under normal conditions, ROS production is regulated, and some of them serve beneficial functions such as serving as critical regulators of pathophysiological and physiological consequences [64]. They are generated in response to physiological signals as signaling molecules, which are required to regulate processes such as inflammation [64]. Alternatively, uncontrolled oxidant generation induces oxidative stress, which directly impacts cellular activities and leads to the development of chronic diseases [65]. Endogenous antioxidants such as GSH and GST are often effective in preventing ROS and RNS production [66]. The first line of antioxidant, GSH, catalytically scavenges free radicals (O_2_ and H_2_O_2_). In the presence of GSH, endogenous glutathione is oxidized to produce water, and H_2_O_2_ is reduced [67]. Cellular dysfunction and excessive pathological conditions, such as the breakdown of bone and cartilage, are caused by an imbalance in this process during the exacerbated cell response [68]. In addition, injection of CFA causes significant production of ROS and FR at the site of inflammation in rats [54]. Our results demonstrated that the MDA level was significantly increased and GSH and GST were significantly decreased in the arthritic group as compared to normal rats, which is consistent with Ahmed et al. [69]. An increased number of leukocytes in the blood and inflamed areas, which increases rates of LPO production and suppresses the antioxidant defense system, may be the cause of elevated MDA levels. Nonetheless, Nano ZT/Vit B12 treatment significantly reduced the MDA level and increased the antioxidants. Our findings are consistent with the findings of Pavelic et al. [14]. Furthermore, Nano ZT/Vit B12 therapeutic agents’ antioxidant defense mechanism against oxidative stress was primarily achieved through the activation of an antioxidant enzyme, which played a critical role in reducing oxidative stress by eliminating H_2_O_2_, which is used as an oxidative stimulus for cell death. This conclusion is consistent with the findings of Pavelic et al. [14] in their study of the use of ZT in the treatment of bone disorders. According to our findings, Nano ZT/Vit B12 was effective in boosting antioxidant defenses at the expense of oxidative stress in tissues, thereby inhibiting subsequent inflammatory processes.

Histopathological examination demonstrated that rats in the arthritic group showed a severe case of inflammation as evidenced by leukocyte infiltration, synovial hyperplasia, cartilage deterioration, and histologically confirmed bone resorption. Animals given Nano ZT/Vit B12, on the other hand, had milder stages of these lesions and significantly less joint damage. Early stages of these lesions were also demonstrated with normal joint space, minimal leukocyte infiltration, and cartilage that appeared undamaged. When compared to arthritic control rats, a high level of protection was also provided against such deformation. The Nano ZT/Vit B12 reduced cartilage deterioration, suppressed pannus development, and decreased synovitis. As a result, these findings clearly demonstrated Nano ZT/Vit B12’s efficacy in decreasing inflammatory responses within tissues, as well as suppressed inflammatory reactions and paw swelling caused by arthritis. Thus, these findings demonstrated the anti-inflammatory activity of Nano ZT/Vit B12 treatments as therapeutic targets for RA due to their inhibitory effects in repressing inflammation inside tissues and constraining the swelling features of arthritis. Collectively, our finding revealed and explained the efficacy of Nano ZT/Vit B12 as anti-arthritic, anti-inflammatory, and antioxidant agents in the treatment of RA.

## 5. Conclusions

**Figure 13:**
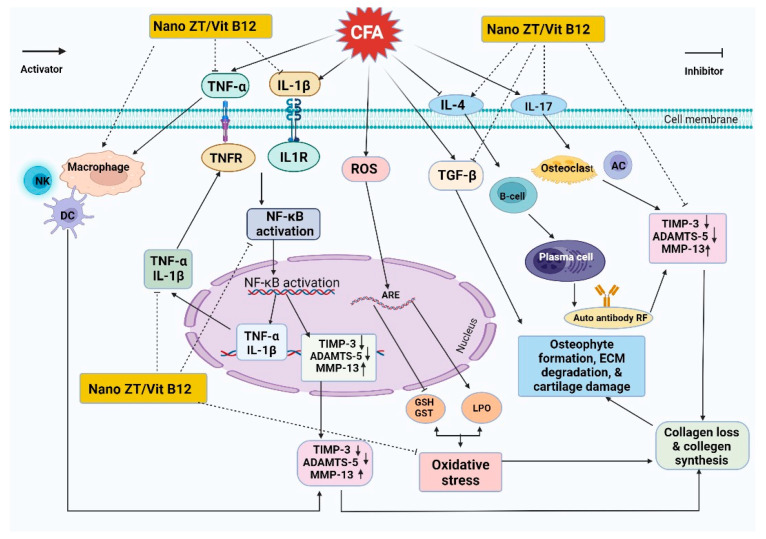
Schematic figure showing the mechanisms of action of Nano ZT/Vit B12 in arthritic rats.

In the current study, the newly synthesized Nano ZT/Vit B12 was shown to have anti-arthritic, anti-inflammatory, and antioxidant effects in CFA-induced arthritis. The anti-arthritic effect of Nano ZT/Vit B12 may be supported by the suppression of inflammation, the reduction in oxidative stress, the enhancement of the antioxidant defense system, the increase in anti-inflammatory markers such as TIMP-3 and IL-4, and the decrease in ADAMTS-5, TGF-β, and MMP-13 levels (Figure 13). In addition, Nano ZT/Vit B12 improved the histopathologic implications of the articular joints of CFA-induced arthritic rats. Nano ZT/Vit B12 may provide an additional effective treatment option for RA in the future. However, more research is needed to understand the mechanisms underlying Nano ZT/Vit B12’s anti-rheumatic action.

## Figures and Tables

**Table 1:** Primers used for qRT-PCR.

Genes.	GenBank Accession Number	Sequence (5′–3′)
TGF-β	XM_032894155.1	F: GACTCTCCACCTGCAAGACC R: GGACTGGCGAGCCTTAGTTT
GAPDH	XM_017592435.1	F: CACCCTGTTGCTGTAGCCATATTC R: GACATCAAGAAGGTGGTGAAGCAG

**Table 2:** Ankle histological lesion scores in the normal control, arthritic, and arthritic with Nano ZT/Vit B12 groups.

	**Cavity**	**Synovial Lining**	**Inflammatory Infiltrate**	**Blood Vessels**	**Pannus**	**Articular Cartilage**	**Menisci**
**Normal**	**0**	**0**	**0**	**0**	**0**	**0**	**0**
**Arthritic**	**0**	**++**	**++**	**0**	**++**	**++**	**++**
**Arthritic + Nano ZT/Vit B12**	**0**	**0**	**+**	**0**	**+**	**0**	**0**
**Cavity:**							
**0:** Average **+:** Narrow **++:** Very narrow							
**Synovial lining:**							
**0:** Average/intact **+:** Thickened/hyperplastic **++:** Necrotic/ulcerated							
**Inflammatory infiltrate:**							
**0:** No **+:** Scattered/mild **++:** Moderate/marked/with excess fibroblasts							
**Blood vessels:**							
**0:** Average **+:** Mildly dilated/congested **++:** Markedly dilated/congested							
**Pannus:**							
**0:** No **+:** Small/large non-destructing **++:** Large destructing/with fibrous bands							
**Articular cartilage:**							
**0:** Average **+:** Mildly destructed/thickened **++:** Markedly destructed							
**Menisci:**							
**0:** Average **+:** Mildly destructed **++:** Markedly destructed							

## Data Availability

All data can be found in the article.

## Supplementary Materials

The following supporting information can be downloaded at: https://www.mdpi.com/1424-8247/16/2/285/s1, Figure S1: Presented the hydrodynamic particle size and the zeta potential of the prepared Nano ZT (A and B) and Nano ZT/VB12 (C and D) respectively; Table S1: Applied conditions of the ball milling process for the preparation of Nano ZT; Table S2: BJH pore size distribution adsorption of the prepared Nano ZT/Vit B12.
