# Correction: Belal et al. Therapeutic Potential of Zeolites/Vitamin B12 Nanocomposite on Complete Freund’s Adjuvant-Induced Arthritis as a Bone Disorder: In Vivo Study and Bio-Molecular Investigations. *Pharmaceuticals* **2023**, *16*, 285

**DOI:** 10.3390/ph17091172

**Published:** 2024-09-05

**Authors:** Amany Belal, Rehab Mahmoud, Mohamed Taha, Fatma Mohamed Halfaya, Ahmed Hassaballa, Esraa Salah Elbanna, Esraa Khaled, Ahmed Farghali, Fatma I. Abo El-Ela, Samar M. Mahgoub, Mohammed M. Ghoneim, Mohamed Y. Zaky

**Affiliations:** 1 Department of Pharmaceutical Chemistry, College of Pharmacy, Taif University, Taif 21944, Saudi Arabia; 2 Department of Chemistry, Faculty of Science, Beni-Suef University, Beni-Suef 62511, Egypt; radwaraft@yahoo.com; 3 Materials Science and Nanotechnology Department, Faculty of Postgraduate Studies for Advanced Sciences, Beni-Suef University, Beni-Suef 62511, Egypt; mtaha@psas.bsu.edu.eg (M.T.); esraa.salah@psas.bsu.edu.eg (E.S.E.); d_farghali@yahoo.com (A.F.); miramar15@yahoo.com (S.M.M.); 4 Department of Surgery, Anesthesiology and Radiology, Faculty of Veterinary Medicine, Beni-Suef University, Beni-Suef 62511, Egypt; fatma.halfaia@vet.bsu.edu.eg; 5 Nutrition and Food Science, College of Liberal Arts and Sciences, Wayne State University, Detroit, MI 48202, USA; eu3412@wayne.edu; 6 ZeroHarm L.C., Farmington Hills, Farmington, MI 48333, USA; 7 Biotechnology Department, Faculty of Postgraduate Studies for Advanced Sciences, Beni-Suef University, Beni-Suef 62511, Egypt; esraa899@yahoo.com; 8 Department of Pharmacology, Faculty of Veterinary Medicine, Beni-Suef University, Beni-Suef 62511, Egypt; fatma.aboel3la@vet.bsu.edu.eg; 9 Department of Pharmacy Practice, College of Pharmacy, AlMaarefa University, Ad Diriyah 13713, Saudi Arabia; mghoneim@mcst.edu.sa; 10 Pharmacognosy and Medicinal Plants Department, Faculty of Pharmacy, Al-Azhar University, Cairo 11884, Egypt; 11 Molecular Physiology Division, Zoology Department, Faculty of Science, Beni-Suef University, Beni-Suef 62521, Egypt; 12 Department of Oncology and Department of Biomedical and Clinical Sciences, Faculty of Medicine, Linköping University, 581 83 Linköping, Sweden

In the original publication [1], reference 20 had the wrong citation by mistake. The correct citation for reference 20 [2], has now been added in 2. Materials and Methods, 2.2.2. Nano ZT/Vit B 12 Characterization.

The authors state that the scientific conclusions are unaffected. This correction was approved by the Academic Editor. The original publication has also been updated.
