# Supplementary material for: Therapeutic Potential of Zeolites/Vitamin B12 Nanocomposite on Complete Freund’s Adjuvant-Induced Arthritis as a Bone Disorder: In Vivo Study and Bio-Molecular Investigations
Source: Pharmaceuticals (Basel). 2023 Feb 13;16(2):285. doi: 10.3390/ph16020285 (PMC9964923; doi:10.3390/ph16020285)
Supplement: Supplementary file 1 [file pharmaceuticals-16-00285-s001.zip › pharmaceuticals-2077204-supplementary.pdf]

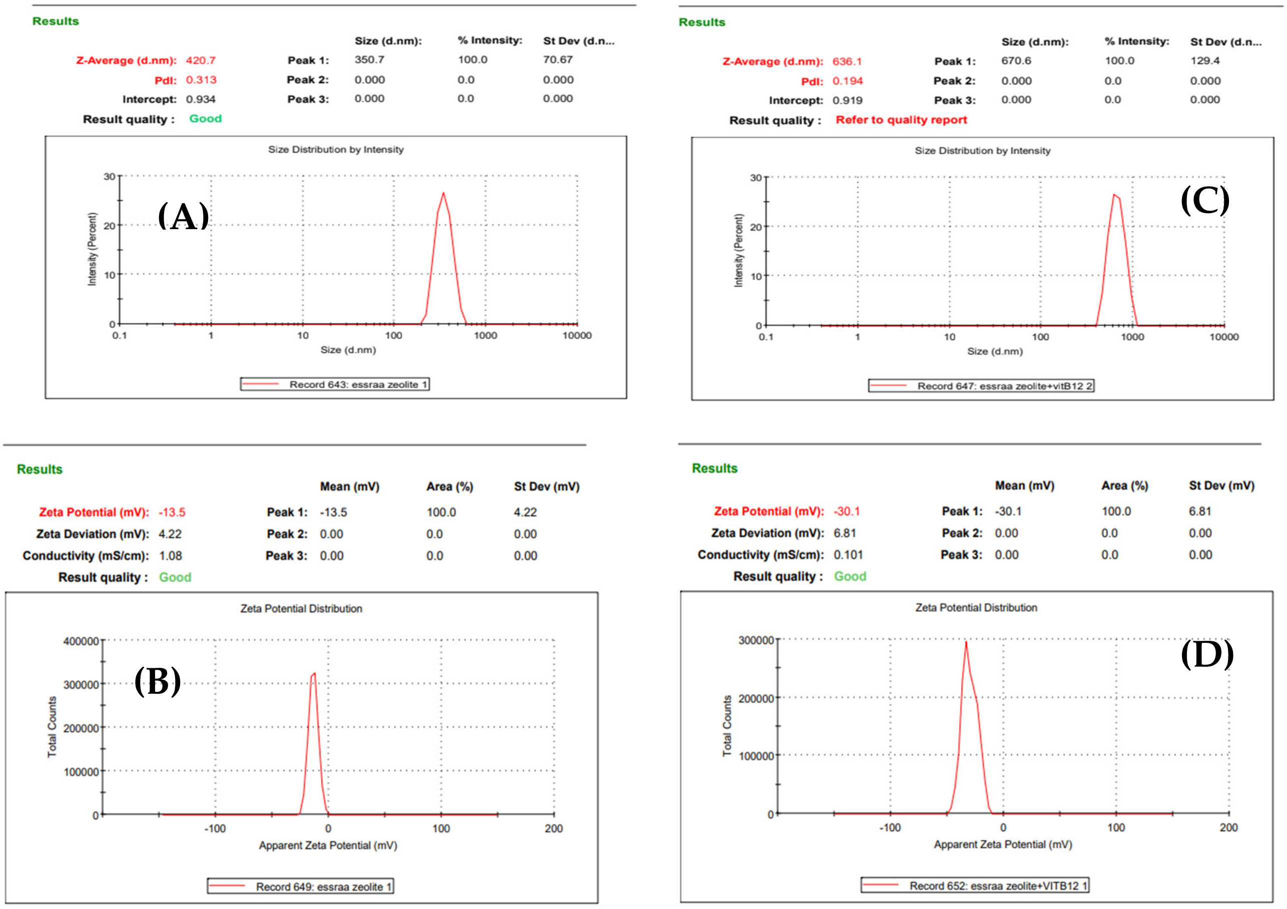

Figure S1. presented the hydrodynamic particle size and the zeta potential of the prepared Nano ZT (a&b) and Nano ZT/VB12 (c&d) respectively.

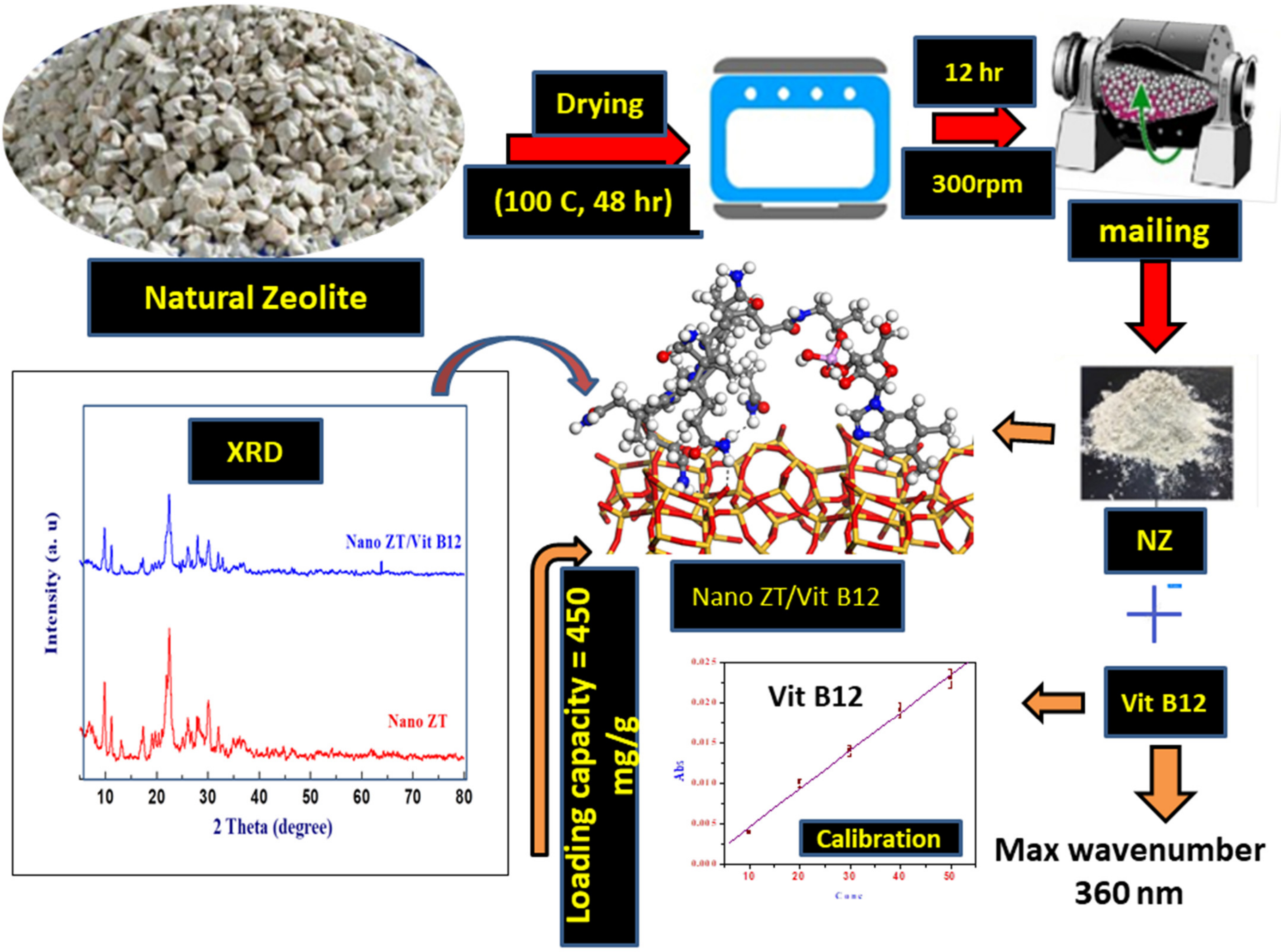

Figure S2. Preparation of Nano ZT/Vit B 12.

Table S1. Applied conditions of the ball milling process for the preparation of Nano ZT.

Process Condition	Description
Balls diameters	Ranged from 1.6 to 1.9 cm
Vessel diameter	7.8 cm
Materials of vessel	Stain steel
Materials of used balls	Porcelain
Ball/Natural-zeolite mass ratio	15:1
Speed	300 rpm
Time	12 h

Table S2. BJH pore size distribution adsorption of the prepared Nano ZT/Vit B12.

Table - BJH Pore Size Distribution Adsorption

radius nm	Pore Volume cc/g	Pore Surf. Area m^2/g	dV(r) cc/nm/g	dS(r) $m^2/nm/g$	dV(log r) cc/g	dS(log r) m^2/g
1.92090	8.025859e-03	8.356359e+00	1.613582e-02	1.680029e+01	7.096870e-02	7.389115e+01
2.56579	1.837888e-02	1.642640e+01	1.306551e-02	1.018439e+01	7.657298e-02	5.968760e+01
3.70896	3.241140e-02	2.399323e+01	9.392978e-03	5.065024e+00	7.912127e-02	4.266497e+01
6.51695	5.681940e-02	3.148384e+01	5.921321e-03	1.817206e+00	8.580859e-02	2.633397e+01
60.4979	1.400603e-01	3.423570e+01	8.016277e-04	2.650100e-02	7.449183e-02	2.462624e+00
